# Exosomes From Packed Red Cells Induce Human Mast Cell Activation and the Production of Multiple Inflammatory Mediators

**DOI:** 10.3389/fimmu.2021.677905

**Published:** 2021-05-06

**Authors:** Xiaobin Fang, Jingyi Li, Xuechao Hao, Weiyi Zhang, Jie Zhong, Tao Zhu, Ren Liao

**Affiliations:** ^1^ Department of Anesthesiology, West China Hospital, Sichuan University & The Research Unit of West China (2018RU012), Chinese Academy of Medical Science, Chengdu, China; ^2^ Department of Dermatovenereology, West China Hospital of Sichuan University, Chengdu, China

**Keywords:** exosome, inflammatory mediators, human mast cell line, MAPK cell signaling pathway, toll-like receptor

## Abstract

Most blood transfusion-related adverse reactions involve the immunologic responses of recipients to exogenous blood components. Extracellular vesicles isolated from packed red cells can affect the recipient’s immune system. Mast cells are traditionally known as effector cells for allergic transfusion reactions. However, growing evidence supports the notion that activated mast cells might disturb host innate immunologic responses. Exosomes are a type of extracellular vesicle. To determine the effect of exosomes on mast cells, we enriched exosomes derived from volunteer plasma (EXs-nor) and packed red cells (EXs-RBCs) using ultracentrifugation and incubated them with a human mast cell line (HMC-1). We found that EXs-RBC exposure increased the expression of tryptase-1 and prostaglandin D2, the production of multiple inflammatory mediators, and the levels of Toll-like receptor-3 (TLR-3) and phospho-mitogen-activated protein kinase (MAPK) in HMC-1 cells. MAPK inhibitors (SB203580, PD98059, and SP600125) and a TLR-3/dsRNA complex inhibitor reduced the EXs-RBC-stimulated production of inflammatory mediators in HMC-1 cells, whereas the TLR-3 agonist [poly (A:U)] elevated the production of these mediators. These results indicate that EXs-RBCs activate HMC-1 cells and elicit the production of multiple inflammatory mediators, partly *via* the TLR-3 and MAPK pathways. Mast cells activated by EXs-RBCs exhibit complex inflammatory properties and might play a potential role in transfusion-related adverse reactions.

## Introduction

Blood transfusion is an essential therapeutic method in clinical practice, and packed red cell (RBC) transfusion is the primary blood transfusion administration used to treat hemorrhage and improve tissue oxygenation (1). Although RBC transfusion is lifesaving, it is associated with many transfusion-related adverse reactions (2), most of which involve immunologic responses of recipients to exogenous blood components. However, the blood components that attack the recipient’s immune response and the underlying mechanism of this process are mostly unknown.

Recently, the potential immunologic effect of extracellular vesicles from RBCs has attracted growing interest (3). Extracellular vesicles are small double-membrane vesicles that can be secreted by RBCs and increase in number with storage time. Extracellular vesicles in RBCs possess pro-inflammatory (4) and immunosuppressive abilities (5) and can activate monocytes (6) and complement systems (7). Extracellular vesicles can be classified into three types according to size. Particularly, exosomes (EXs), a type of extracellular vesicle with a diameter ranging between 30 and 150 nm, contain RNA, DNA, and protein, which might participate in many critical physiological and pathological processes (8).

Mast cells are robust and rapidly activated immune cells with tissue-resident and hematopoietic origins (9). Mast cells are best known for their role in allergic reactions. The primary mechanism of allergic reactions involves mast cell activation by IgE *via* the high-affinity receptor FcϵRI and the release of histamine by the activated mast cells. One example of transfusion-related events that might occur *via* this mechanism is the allergic transfusion reaction (10, 11). In addition to their critical role in allergic reactions, mast cells can be activated *via* the IgE-independent pathway to produce various cytokines, which interact with other immune cells (12–14). Activated mast cells can disturb the host immunologic response to exogenous stimuli and might be detrimental to the host (15). Importantly, mast cells can also be the target cells of extracellular vesicles derived from platelets and activated T cells (16–18). We hypothesized that EXs isolated from RBCs (herein, EXs-RBCs) would elicit mast cell activation based on previous findings (12–18).

If mast cells can respond to EXs-RBCs, identifying the holistic inflammatory patterns of these mast cells responding to EXs-RBCs is essential for discovering their potential role in transfusion-related adverse events. The activation of mast cells *via* the Toll-like receptor-3 (TLR-3) and mitogen-activated protein kinase (MAPK) pathways has been investigated (19, 20). TLR-3 agonists can mediate the immune response in a mouse RBC transfusion model (21). Therefore, we investigated whether TLR-3 and MAPK pathways are involved in mast cell activation after EXs-RBC stimulation. This study thus aimed to determine whether EXs-RBCs can elicit mast cell activation and explore the inflammatory patterns of mast cells responding to EXs-RBCs, as well as the involvement of TLR-3 and MAPK signaling pathways.

## Materials and Methods

**Figure 1:**
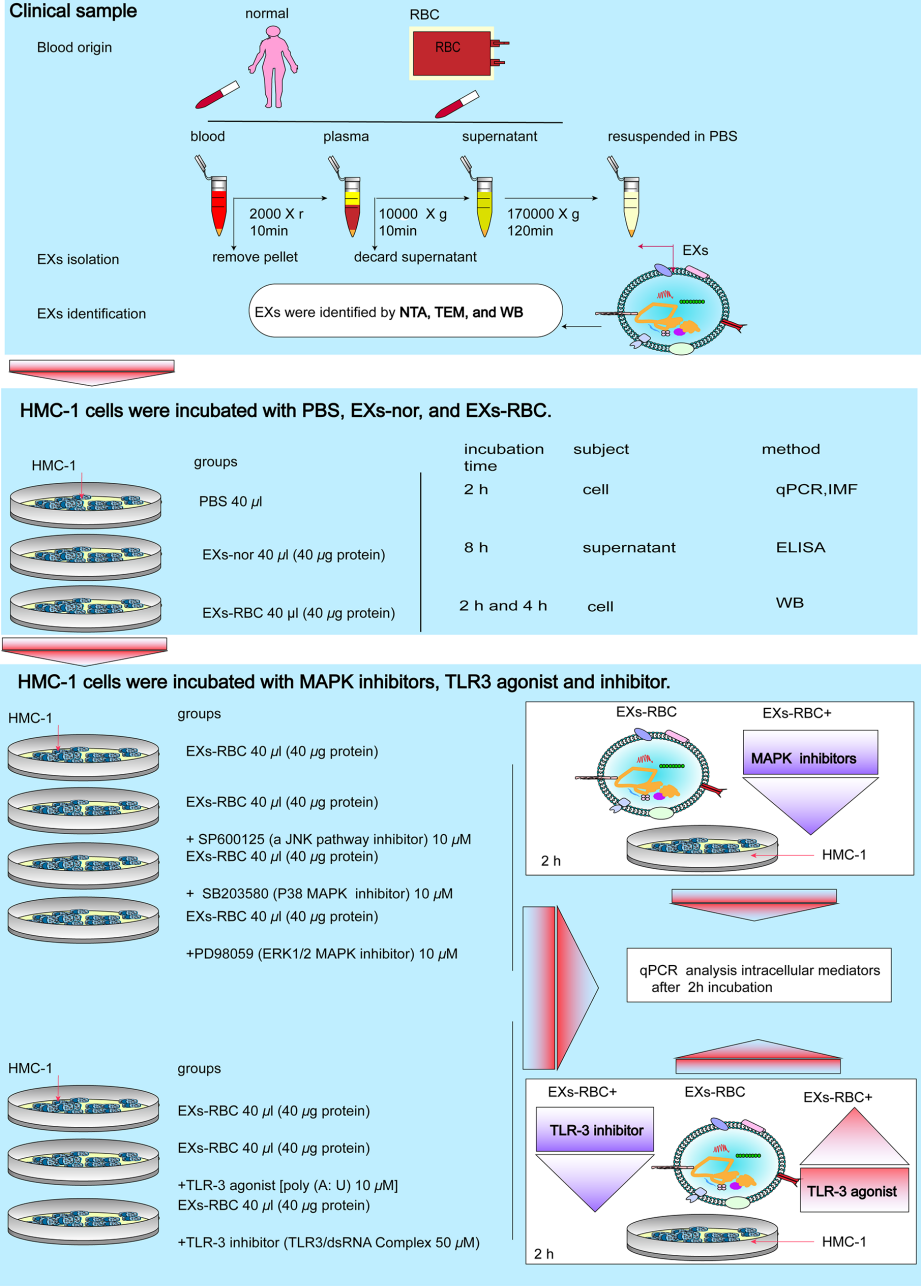
Flowchart of this study. EXs, exosomes; RBC, red cell units; EXs-nor, exosomes from normal volunteer plasma; EXs-RBC, exosomes from red cell units; WB, western blotting analysis; IMF, immunofluorescence; qPCR, quantitative real-time PCR; ELISA, enzyme-linked immunosorbent assay; NTA, nanoparticle tracking analysis; TEM, transmission electron microscopy.

The study protocol was approved by the Biological-Medical Ethical Committee of the West China Hospital of Sichuan University (Chengdu, Sichuan, China) on July 18, 2019 (Ethical approval number: 2019494). Details are presented as **Supplementary Data 1**. The flowchart is presented in **Figure 1**.

### Isolation and Identification of EXs

Healthy volunteers 16 to 60 years of age were recruited; volunteers with a history of allergic diseases were excluded. Blood samples were collected from four healthy volunteers after patient consent was obtained, and four bags of stored red cells (8 mL each) of A, B, O, and AB types were obtained. Subject information is presented in **Supplementary Table 1**. EXs were extracted from platelet-free plasma of healthy volunteers (herein, EXs-nor) and stored red cells using ultracentrifugation. The characterization of EXs was performed using transmission electron microscopy (TEM), nanoparticle tracking analysis (NTA), and western blot analysis. The protein concentrations of the EXs were quantified using the Pierce™ BCA Protein Assay kit (Thermo Fisher Scientific Inc.) as previously described (22). All EXs were diluted to 1 µg protein/µL with PBS.

### Culture and Treatment of HMC-1 cells

HMC-1 cells were cultivated under the conditions of 95% oxygen/5% CO_2_ and 37°C and grown in IMDM (SH30228.01, Gibco), consisting of streptomycin (100 μg/mL)/penicillin (100 U/mL) and 10% heat-inactivated fetal bovine serum. Before the experiments, HMC-1 cells were cultivated with the serum-free basal medium and washed twice using PBS, then fresh IMDM (1 mL) was add in each culture dish. At a 1,000,000 cells/mL concentration, HMC-1 cells received diverse treatments. In the first part of the experiment, 40 μL PBS, 40 μL EXs-nors, and 40 μL EXs-RBCs were used to investigate the effect of exosomes on mast cells. In the second part, 40 μL EXs-RBCs plus SP600125 (a JNK pathway inhibitor) at 10 μM, 40 μL EXs-RBCs plus SB203580 (P38 MAPK selective inhibitor) at 10 μM, 40 μL EXs-RBCs plus PD98059 (ERK1/2 MAPK inhibitor) at 10 μM, 40 μL EXs-RBCs plus poly (A:U) at 10 μM, and 40 μL EXs-RBCs plus TLR-3/dsRNA complex at 50 μM were employed to explore the possible functions of MAPKs pathways and TLR-3.

We chose an amount of 40 micrograms/mL (40µL EX in 1mL medium) because of the visible cell death observed with 100, 70, and 50 micrograms/mL EX treatment (data not shown). The time-frame of the study was based on our preliminary experiments and a previous study using a mouse mast cell line (23). Forty microliters (40 µg protein) of EXs was used in all of our experiments.

### HMC-1 Cell Incubation

We focused on the expression of mast cell activated markers, tryptase-1 and prostaglandin D2 (PGD-2), the inflammatory pattern of HMC-1 cells responding to EXs-RBCs, and the activation of TLR-3 and MAPK pathways. After incubation with PBS, EXs-nors, and EXs-RBCs, HMC-1 cell culture plates were centrifugated for 10 min at 450 × *g* at 4°C. At 2 h after incubation, the cells were harvested and analyzed using quantitative real-time PCR (qPCR) and Immunofluorescence. After incubation for 2 h and 4 h, cells were collected for western blot analysis. After incubation for 8 h, supernatants were collected and analyzed using enzyme-linked immunosorbent assay.

To evaluate the involvement of MAPK signaling pathways in HMC-1 cell activation after EXs-RBC stimulation, we incubated HMC-1 cells with PBS, EXs-nors, EXs-RBCs, EXs-RBCs + SP600125 at 10 µM, EXs-RBCs + SB203580 at 10 µM, and EXs-RBCs + PD98059 at 10 µM. After 2 h, cells were collected for qPCR analysis. To investigate the effect of TLR-3 on the response of HMC-1 cells to EXs-RBCs, HMC-1 cells were incubated with PBS, EXs-nors, EXs-RBCs, EXs-RBCs plus poly (A:U; 10 µM), and EXs-RBCs + TLR-3/dsRNA complex (50 µM) for 2 h. Cells were collected for qPCR analysis. Details of qPCR, western blotting, and ELISA are presented in **Supplementary Data 1**.

### HMC-1 Cell Viability

HMC-1 cells (10,000 cells/mL) were seeded in 96-well plates and administered various treatments. After 24 h of treatment, mentioned previously herein, 10 µL of CCK8 (Dojindo, Japan) solution was added into the wells, and the cells were cultured for a further 90 min at 37°C with 5% CO_2_. The optical density (OD) at 450 nm was measured using a spectrophotometer. Relative cell viability was equal to the ratio between the OD of treatment groups and the OD of the blank group.

### qPCR

For qPCR, total RNA was extracted using the TRizol reagent. A reverse transcription kit (Bio-Rad, Britain) was used to synthesize cDNA. cDNA expression was analyzed using Maxima™ SYBR Green qPCR Master Mix (Fermentas, Vilnius, Lithuania) according to the manufacturer’s introductions. Relative mRNA expression was normalized to that of 18S RNA. The qPCR primers used are listed in **Supplementary Table 2**. In the first part of the experiment, qPCR was employed to evaluate the expression of inflammatory mediators in HMC-1 cells respond to EXs-RBC. In the second part, qPCR was used to evaluate the regulation of MAPK and TLR-3 on inflammatory mediators’ production in HMC-1 cells induced by EXs-RBC.

### Western Blotting

For western blotting, cells were collected, washed twice, mixed with RIPA buffer, and centrifuged. Total protein concentration was normalized using the BCA assay. Mixtures were electrophoresed and transmitted to the PVDF membrane. After blocking for 1 h by 5% milk, membranes were incubated at 4°C overnight with human antibodies against lamin B1, α-tubulin, TLR-3, Tryptase-1, phosphorylated-JNK, phosphorylated-P38 (Thr180/Tyr182), phosphorylated-ERK1/2(p44/42), total JNK, P38 and ERK1/2, and then secondary antibodies (HRP-conjugated). Blots were visualized as the procedure mentioned above (subsection 2.1). The relative amount of protein was quantified by the ratio to α-tubulin or lamin B1. Western blotting was performed to determine the expression of tryptase-1, PGD-2, TLR-3, and MAPK in HMC-1 cells induced by EXs-RBCs.

### Immunofluorescence

For immunofluorescence, HMC-1 was resuspended, and 100 µL solution was transferred into 96-well plates, which have been pretreated with polylysine for 30 min. HMC-1 were treated by PBS, EXs-nor, and EXs-RBC for 8 h and subsequently fixed with 4% paraformaldehyde for 10 min. Samples were blocked with donkey serum and incubated overnight with anti-Tryptase-1 antibodies, followed by donkey-anti-rabbit IgG (H+L) Highly Cross-Adsorbed secondary antibodies for 2 h. Nuclei were counterstained with DAPI. Images were visualized using an automatic positive fluorescence microscope (Zeiss, German). Immunofluorescence was conducted to determine the expression of tryptase-1 in HMC-1 cells induced by EXs-RBCs.

### ELISA

For ELISA, the concentrations of IL-6, TNF-α, IL-4, CCl-2, CXCL-1, CXCL-5, LTB-4, and VEGF in the supernatant were measured using ELISA according to the manufacturer’s instruction. Mediators’ concentrations are expressed as pg/mL of protein. ELISA was applied to determine the expression of inflammatory mediators in supernatants after HMC-1 cell incubated with EXs-RBCs.

### Statistical Analysis

Data are presented as the mean ± SEM. SPSS v20.0 (Chicago, IL, USA) was used for data analysis. Data on EX marker proteins were analyzed using unpaired Student’s *t*-tests. HMC-1 cell viability was analyzed by one-way ANOVA. The levels of multiple inflammatory mediators in HMC-1 cells and the supernatant and the activation of intracellular MAPK and TLR3 were analyzed using one-way ANOVA with Dunnett’s multiple comparisons tests. *P* < 0.05 was considered statistically significant.

## Results

### Characterization of EXs-Nors and EXs-RBCs

**Figure 2:**
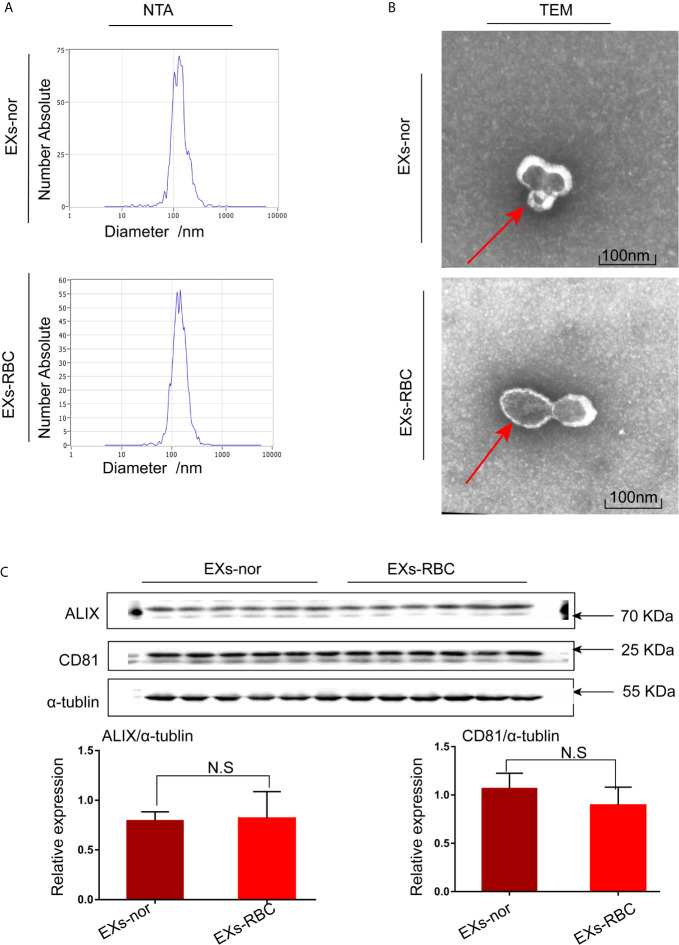
Exosomes (EXs) were identified by nanoparticle tracking analysis (NTA), transmission electron microscopy (TEM), and western blotting analysis. Morphology and size of EXs-nor and EXs-RBC were analyzed using NTA and TEM. **(A)** Representative picture of NTA; 50 µL of EXs (four samples, two from EXs-nor, two from EXs-RBC) was diluted in 50 mL PBS, and NTA was performed. **(B)** Representative picture of TEM; 50 µL EXs was stained with 2% phosphotungstic acid for TEM. TEM revealed EXs that were membrane-bound; red arrow points to the membrane of EXs. Scale bars: 100 nm. **(C)** Western blotting for CD81 and ALIX, marker proteins of EXs. Six samples (3 for EXs-nor, 3 for EXs-RBC) with the same protein concentration were used in duplicate. Blots were quantified by Image J software and standardized to α-tubulin expression. Unpaired student t-tests were performed. There was no statistical difference in the expression of CD81 and ALIX between EXs-RBC and EXs-nor groups. NS, no statistical difference; EXs-nor, exosomes from normal volunteer plasma; EXs-RBC, exosomes from red cell units.

According to NTA results, the diameter of EXs-nors were approximately 132.8 nm, and EXs-RBC were 136 nm (**Figure 2A**). TEM revealed that EXs contained morphologically distinct membrane-bound particles that were approximately 100 nm in diameter (**Figure 2B**). EXs-nor and EXs-RBC both expressed the marker proteins CD81 and ALIX of exosomes (**Figure 2C**).

### Viability of HMC-1 Cells Under Diverse Treatment Conditions

CCK-8 assay results showed no significant change in HMC-1 cell viability after treatment for 24 h (*p* > 0.05, data not shown), indicating that the experimental outcomes were not associated with HMC-1 cell viability under various challenges.

### Increased Expression of Tryptase-1 and PGD-2 in HMC-1 Cells Induced by EXs-RBCs

**Figure 3:**
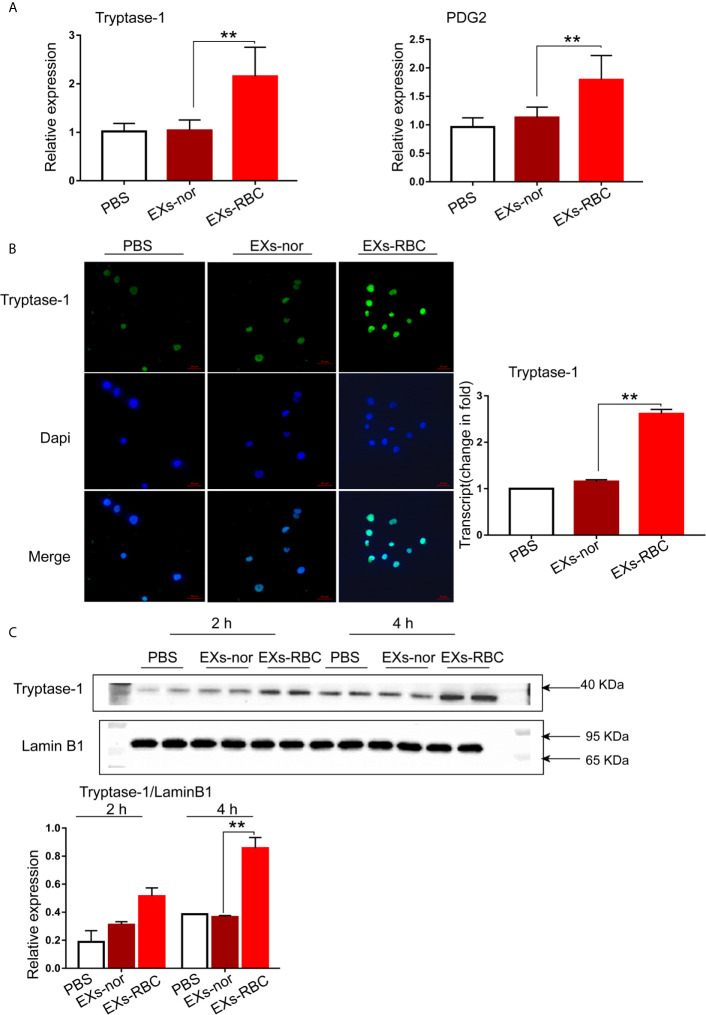
Activation of HMC-1 cells induced by EXs-RBCs. HMC-1 cells were cultivated with PBS, EXs-nor, and EXs-RBC for 2 h, and qPCR and Immunofluorescence were performed. At 2 h and 4 h after incubation, western blotting was employed. Data were from 2–4 independent trials. ANOVA and multiple comparisons were used to measure the differences between EXs-nor and EXs-RBC groups. * indicates statistical difference between groups (**P < 0.01). **(A)** The expression of *tryptase-1* and *PGD-2* mRNA in HMC-1 cells increased in the EXs-RBC group. **(B)** Left pictures are the representative panels of Immunofluorescence. Right panel: immunofluorescence results were quantified with Image J software. **(C)** Representative western blotting images (upper panels). Blots were quantified using Image J (lower panel). EXs-nor, exosomes from normal volunteer plasma; EXs-RBC, exosomes from red cell units.

Compared to that in the EXs-nor group, the expression of *tryptase-1* and *PGD-2* mRNA in HMC-1 cells increased significantly in the EXs-RBC group (*p* < 0.05; **Figure 3A**). The marked augmentation of Immunofluorescence also suggested increased expression of tryptase-1 in the EXs-RBC group (**Figure 3B**). According to western blotting analysis, the expression of tryptase-1 in the EXs-RBC group increased after 2 h and showed a statistical difference after 4 h (**Figure 3C**). The increased expression of tryptase-1 and PGD-2 indicated that EXs-RBCs can activate HMC-1.

### EXs-RBC Exposure Increases the Production of Multiple Inflammatory Mediators in HMC-1 Cells

**Figure 4:**
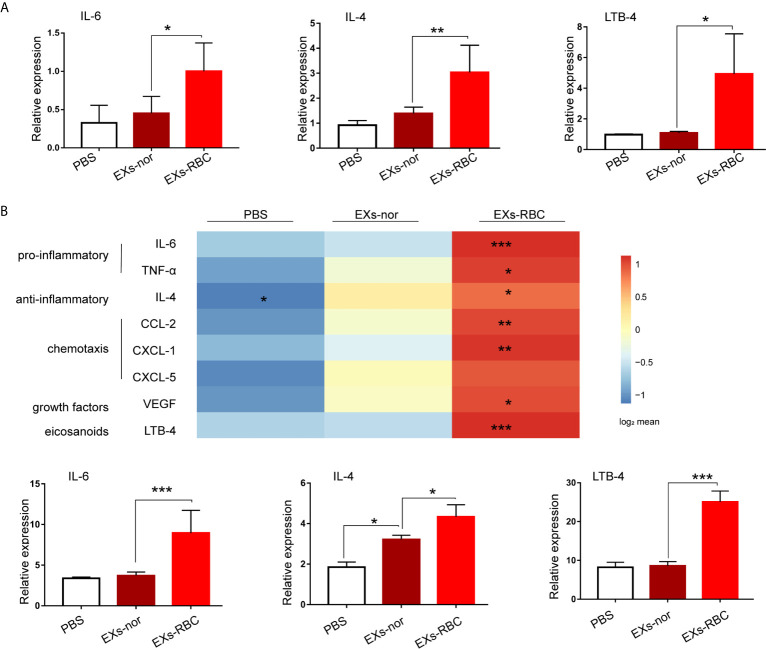
EXs-RBC exposure upregulates the expression and secretion of multiple mediators in HMC-1 cells. HMC-1 cells were cultivated with PBS, EXs-nor, and EXs-RBC. Cells were collected for qPCR at 2 h. Supernatants were collected for ELISA at 8 h. Data were from 2–4 independent trials. ANOVA and multiple comparisons were applied to test the differences between EXs-nor and EXs-RBC groups. * means a statistical difference between groups. (*P < 0.05; **P < 0.01;***P < 0.001). Here, the results of IL-6, IL-4, and LTB-4 are shown, whereas the results for other inflammatory mediators are shown in **Supplementary Figure 1**. **(A)** qPCR results. **(B)** ELISA outcomes. Upper panel: results of mediator concentrations in supernatants stimulated by PBS, EXs-nor, and EXs-RBC; data are shown using the method of the heat picture according to the log_2_ transformed average value from 2–4 independent trials. EXs-nor, exosomes from normal volunteer plasma; EXs-RBC, exosomes from red cell units.

Compared to those in the EXs-nor group, the EXs-RBC group showed increased levels of *IL-6*, *TNF-α*, *IL-4*, *INF-γ*, *CCl-2*, *CXCL-1*, *PAF*, *LTB-4*, and *VEGF* mRNA in HMC-1 cells (**Figure 4A** and **Supplementary Figure 1A**). Further, compared to levels in the EXs-nor group, the EXs-RBC group showed increased expression of IL-6, TNF-α, IL-4, CCl-2, CXCL-1, PAF, LTB-4, and VEGF in the supernatant (**Figure 4B** and **Supplementary Figure 1B**).

### EXs-RBC Exposure Increases the Phosphorylation of MAPKs and *TLR-3* mRNA in HMC-1 Cells

**Figure 5:**
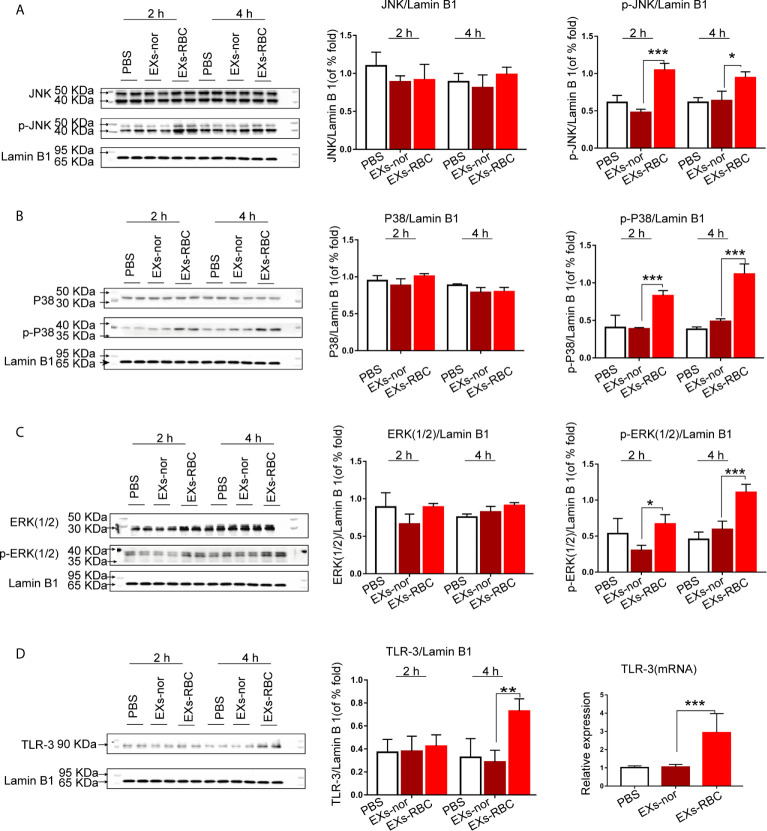
Expression of MAPK pathway and TLR-3 in HMC-1 cells after incubation with PBS, EXs-nor, and EXs-RBC. Cells were collected at 2 h and 4 h. Blots were standardized to lamin B1 expression. Each experiment was employed at least twice. ANOVA and multiple comparisons were performed to compare the differences among groups. * means a statistical difference between two groups (*P < 0.05; **P < 0.01; ***P < 0.001). The left panels are the representative pictures of western blotting, and the right and middle panels are the quantitation of western blotting using Image J software. **(A)** Expression of JNK and phospho-JNK. **(B)** Expression of P38 and phospho-P38. **(C)** Expression of ERK1/2 and phospho-ERK1/2. **(D)** Right panels: cells were collected at 2 h. *TLR-3* mRNA was measured by qPCR. 18S was used for standardization. Left and middle panels: cells were collected at 2 h and 4 h. Blots are standardized to lamin B1 expression. The left panels are the representative pictures of western blotting, and the middle panels are the quantization of western blotting using Image J software. p-JNK, phospho-JNK; p-P38, phospho-P38; p-ERK1/2, phospho-ERK1/2; EXs-nor, exosomes from normal volunteer plasma; EXs-RBC, exosomes from red cell units.

In the EXs-RBC group, the phosphorylation levels of JNK, P38, and ERK1/2 were upregulated 2 h and 4 h after treatment, whereas the level of total MAPKs did not significantly change (**Figures 5A–C**). Compared to that in the EXs-nor group, the level of *TLR-3* mRNA in HMC-1 cells increased in the EXs-RBC groups. Western blotting analysis also revealed a significant increase in TLR-3 expression at 4 h in the EXs-RBC group (**Figure 5D**).

### MAPK Cell Signaling Inhibitors Decrease the Expression of Inflammatory Mediators in HMC-1 Cells After EXs-RBC Incubation

**Figure 6:**
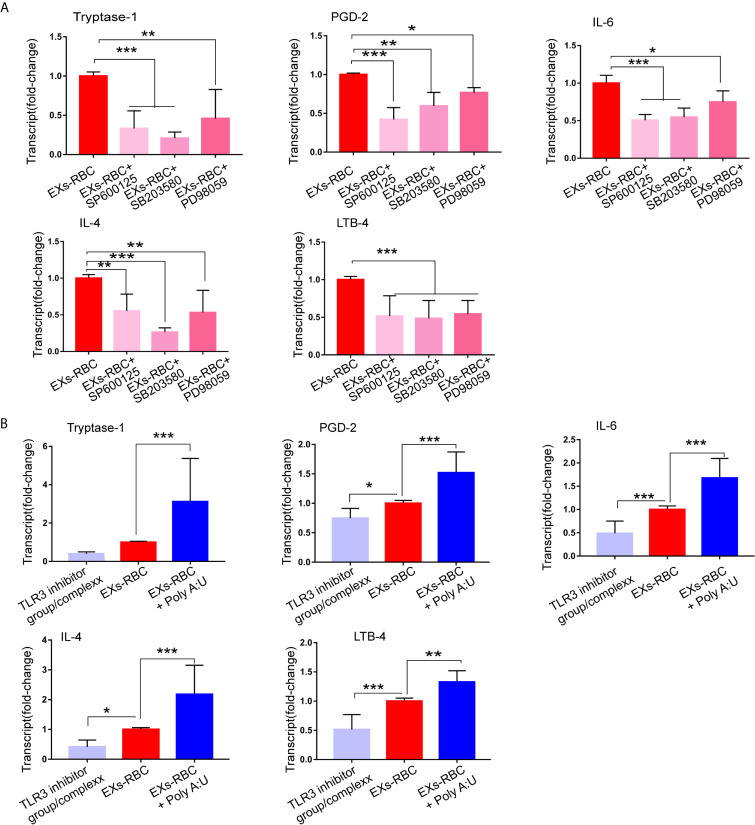
Regulation of the production of inflammatory mediators by MAPK inhibitors and TLR3 inhibitor and agonist. HMC-1 cells were treated with PBS, EXs-nor, EXs-RBC, EXs-RBC plus MAPK inhibitors, EXs-RBC plus TLR3 agonist and inhibit for 2 h. Cells were collected for qPCR analysis. Data of PBS and EXs-nor are not shown. Data were from 2–3 independent experiments. The difference within groups was normalized by values from the EXs-RBC group. ANOVA and Dunnett’s multiple comparisons were used to analyze the differences among groups. * represents a statistical difference (*P < 0.05; **P < 0.01; ***P < 0.001). **(A)** The selective P38 MAPK inhibitor (SB203580), ERK1/2 MAPK inhibitor (PD98059), and JNK pathway inhibitor (SP600125) reduced the expression of mediators in HMC-1 cells induced by EXs-RBCs. **(B)** The TLR-3/dsRNA complex decreased, whereas poly (A:U) increased the expression of mediators in HMC-1 cells responding to EXs-RBCs. Here, show the expression of tryptase-1, PGD-2, IL-6, IL-4, and LTB-4. Other mediators are shown in **Supplemental Content 5**. EXs-nor: exosomes from normal volunteer plasma; EXs-RBC: exosomes from red cell units.

Compared to that in the EXs-RBC group, the expression of tryptase-1, PGD-2, IL-6, TNF-α, IL-4, INF-γ, CXCL-1, CXCL-5, and LTB-4, but not PAF and VEGF, in HMC-1 cells decreased significantly in the EXs-RBC + SB203580, EXs-RBC + SP600125, and EXs-RBC + PD98059 groups (**Figure 6A** and **Supplementary Figure 2A**).

### TLR-3 Agonist Increases, Whereas TLR-3 Inhibitor Decreases, the Expression of Inflammatory Mediators in HMC-1 Cells in Response to EXs-RBCs

We next observed that the expression of *TLR-3* mRNA in HMC-1 cells was reduced in the EXs-RBC + TLR-3 inhibitor group, whereas it was augmented in the EXs-RBC + TLR-3 agonist group (**Supplementary Figure 2B**). These outcomes indicate the efficiency of TLR-3 agonists and inhibitors. Compared to that in the EXs-RBC group, the expression of tryptase-1, PGD-2, IL-6, TNF-α, IL-4, INF-γ, CXCL-1, CXCL-5, and LTB-4, but not PAF and VEGF, in HMC-1 cells increased in the EXs-RBC + TLR-3 agonist group and decreased in the EXs-RBC + TLR-3 inhibitor group (**Figure 6B** and **Supplementary Figure 2C**). These findings indicate the potential involvement of TLR-3 in HMC-1 cell activation by EXs-RBC stimulation.

## Discussion

Overall, we found that (i) the mast cell-activation markers tryptase-1 and PGD-2 and multiple inflammatory mediators were elevated, (ii) TLR-3 and MAPK signaling pathways were activated in HMC-1 cells incubated with EXs-RBCs, and (iii) the modulation of various inflammatory mediators could be enhanced by a TLR-3 agonist and suppressed by TLR-3 and MAPK inhibitors. The interaction between the recipient’s immune system and extracellular vesicles in RBCs might be one of the critical mechanisms for transfusion reactions. In this study, we enriched EXs at the centrifugal speed of 170,000 *g*. NTA and TEM revealed that the diameter of most EXs was less than 200 µm, and these nano-vesicles showed exosome characteristics. These results were in good agreement with a previous study showing that extracellular vesicles in RBCs could be separated using ultracentrifugation (4). RBCs contain EXs from various cell types, such as platelets and white blood cells (3). In this study, we did not identify the cell origin of EXs for two reasons as follows: (i) the valuable contents in EXs from different cells might be the same (8), and (ii) we focused on the pathogenic contents in the total EXs from RBCs rather than those in erythrocyte-derived EXs. Pathogenic contents in our present study refer to EXs-RBC components that might exert mast cell-exciting functions. In addition, we investigated the differential expression of miRNA in EXs and used the total protein content to quantify EXs (8). The storage time of the RBCs used in our study was approximately 15 days. The detectable number of EXs has been reported previously (6, 7). Due to the capricious nature of the contents and properties of EXs under different conditions (24), we used EXs isolated from clinical RBCs as the experimental group and EXs-nors as the control. Exosomes exercise their functions *via* their pathogenic components (8). In our future research, we will analyze the contents of EXs-nor and EXs-RBC to identify the possible pathogenic components.

The increased production of tryptase-1—a unique marker for activated mast cells (25)—and PDG-2—a primary lipid mediator that is *de novo* synthesized when mast cells are activated (26)—in HMC-1 cells incubated with EXs-RBCs suggests that EXs-RBCs could activate mast cells. Mast cell-derived tryptase can activate complement systems (27). PDG-2 is a potent chemotactic factor for receptors in Th2 cells (28). Considering a previous report indicating that extracellular vesicles in RBCs directly affect immune systems (4–7), activated mast cells by EXs-RBCs might amplify the recipient immune response to exogenous blood components. Mast cells can be activated by extracellular particles derived from platelets and T cells (16–18), but the exact components need to be further explored. Based on our finding, the pathogenic contents in EXs should be confirmed.

The upregulation of various inflammatory mediators after the stimulation of mast cells by EXs-RBCs indicates the complex inflammatory properties of mast cells after RBC transfusion. Previous studies (4–7) have demonstrated the effect of EXs-RBC on the production of proinflammatory cytokines in blood cells. Here, we highlighted the eliciting function of EXs-RBC on mast cells, which are potent and multifunctional immune cells. The activation of mast cells is a principal mechanism of allergic reactions during transfusion, and the reported allergens include IgA (29), haptoglobin (30), and C4 (31). IL-6 and IL-4 prime and potentiate antigen/IgE-mediated mast cell degranulation (32, 33). PGD2 induces bronchoconstriction and wheal-and-flare responses in human subjects (28). Considering the findings of our study, EXs-RBCs might contribute to allergic transfusion reactions, at least to some extent, with a synergic or priming effect. Efficient methods that can decrease the pathogenic content in EXs-RBC might lower the incidence of allergic transfusion reactions. Via the production of various inflammatory mediators, mast cells might orchestrate innate and adaptive immunologic responses (15, 34) and play a vital role in lung diseases (35) and brain injury (36). The stabilization of mast cells has been shown to alleviate acute lung injury and intestinal injury in animal models of many critical illnesses, including trauma (37), pancreatitis (38), and lung transplant (39). Transfusion-related lung injury is closely associated with inflammatory mediators produced by the recipient’s deranged immune system (40). Considering that RBC transfusion is usually administered in severe cases and the results of our study, EXs-RBC-induced mast cell activation and the downstream production of inflammatory mediators might contribute to potentially harmful outcomes in recipients after blood transfusion. Owing to the broad-spectrum mediator production ability of mast cells and the activation directly induced by EXs-RBCs, further studies that target mast cells and EXs should be conducted to decrease the occurrence of adverse transfusion events.

The downstream effects on inflammatory mediator production suggest the involvement of TLR-3 and MAPK. The activation of MAPKs has been detected in clinical transfusion (41, 42). MAPK activation is a critical pathway for mast cell activation (43, 44). Previous studies have shown that TLRs and MAPKs are involved in the activation of macrophages (45) and CD4^+^ T cells (20) after RBC transfusion. TLR-3 might participate in the activation of mast cells, which recruit CD8+ T cells (46). Considering the outcomes of our study, TLR-3 and MAPKs could be critical pathways in the modulation of the immune system after transfusion. Notably, modulation of TLR-3 and MAPKs did not affect PAF and VEGF production in HMC-1 cells responding to EXs-RBCs in our study. This might indicate that other receptors and pathways are involved in this process. Mast cells possess various receptors and are involved in complex cell pathways, including the HMGB1/TLR-4 pathway. Their role in resuscitation from hemorrhagic shock has been previously revealed (47). Further investigation might elucidate their roles on the response of mast cells to EXs-RBC.

A major limitation of our study is that the precise cargo in EXs-RBCs that activates mast cells was not identified. Exosomes contain both mRNA and microRNA, and many miRNAs have been shown to activate mast cells (48). Microarray assessments should be conducted to compare the differential expression of miRNA between EXs-RBCs and EXs-nors, and further verification needs to be performed in future studies. Secondly, we did not consider the effect of RBC separation methods on exosomes. Almizraq et al. (49) reported that the blood component separation methods may affect exosome production and functions. In this study, we focused on the function of exosomes derived from clinical RBCs. The present research is a prior model and further studies that include red cells units from a blood bank are needed.

Overall, our findings highlight the potential effect of mast cells on transfusion-related complications and might offer new strategies and methods for reducing the risk of transfusion. In conclusion, we have demonstrated here that exosomes isolated from clinical packed red cells activate the human mast cell line HMC-1 and elicit the production of multiple mediators, partly *via* the TLR-3 and MAPK pathways. These findings suggest that mast cells might exhibit complex inflammatory properties after transfusion and offer new insights into transfusion-related adverse events.

## Data Availability Statement

The raw data supporting the conclusions of this article will be made available by the authors, without undue reservation.

## Ethics Statement

The study protocol was approved by the Biological-Medical Ethical Committee of the West China Hospital of Sichuan University (Chengdu, Sichuan, China) on July 18, 2019 (Ethical approval number:2019494). The patients/participants provided their written informed consent to participate in this study.

## Author Contributions

XF designed and performed these experiments, analyzed data, wrote and finalized the manuscript. JL designed and guided the experiments, and wrote and revised the manuscript. XH and WZ revised the manuscript. JZ collected clinical samples. TZ designed the experiments and revised the manuscript. RL designed the experiments and edited the manuscript. All authors contributed to the article and approved the submitted version.

## Funding

This project was supported by a grant from the National Key R&D Program of China (2018YFC2001800), the Key research and development (R&D) Program of Science & Technology Department of Sichuan Province (2020YFS0195), and the National Natural Science Foundation of China (81502722).

## Conflict of Interest

The authors declare that the research was conducted in the absence of any commercial or financial relationships that could be construed as a potential conflict of interest.

## References

[1] Klein HG Spahn DR Carson JL. Red Blood Cell Transfusion in Clinical Practice. Lancet (2007) 370(9585):415–26. 10.1016/S0140-6736(07)61197-0 17679019

[2] Carson JL Triulzi DJ Ness PM. Indications for and Adverse Effects of Red-Cell Transfusion. N Engl J Med (2017) 377(13):1261–72. 10.1056/NEJMra1612789 28953438

[3] Menocha S Muszynski JA. Transfusion-Related Immune Modulation: Functional Consequence of Extracellular Vesicles? Transfusion (2019) 59(12):3553–5. 10.1111/trf.15461 31322730

[4] Kent MW Kelher MR West FB Silliman CC. The Pro-Inflammatory Potential of Microparticles in Red Blood Cell Units. Transfus Med (2014) 24(3):176–81. 10.1111/tme.12123 PMC4451951 24786047

[5] Sadallah S Eken C Schifferli JA. Erythrocyte-Derived Ectosomes Have Immunosuppressive Properties. J Leukoc Biol (2008) 84(5):1316–25. 10.1189/jlb.0108013 18685086

[6] Danesh A Inglis HC Jackman RP Wu S Deng X Muench MO. Exosomes From Red Blood Cell Units Bind to Monocytes and Induce Proinflammatory Cytokines, Boosting T-Cell Responses in Vitro. Blood (2014) 123(5):687–96. 10.1182/blood-2013-10-530469 PMC3907755 24335232

[7] Zecher D Cumpelik A Schifferli JA. Erythrocyte-Derived Microvesicles Amplify Systemic Inflammation by Thrombin-Dependent Activation of Complement. Arterioscler Thromb Vasc Biol (2014) 34(2):313–20. 10.1161/ATVBAHA.113.302378 24311376

[8] Théry C Witwer KW Aikawa E Alcaraz MJ Anderson JD Andriantsitohaina R. Minimal Information for Studies of Extracellular Vesicles 2018 (MISEV2018): A Position Statement of the International Society for Extracellular Vesicles and Update of the MISEV2014 Guidelines. J Extracell Vesicles (2018) 7(1):1535750. 10.1080/20013078.2018.1535750 30637094 PMC6322352

[9] Prussin C Metcalfe DD. Ige, Mast Cells, Basophils, and Eosinophils. J Allergy Clin Immunol (2006) 117(2):S450–6. 10.1016/j.jaci.2005.11.016 16455345

[10] Abe T Matsumoto C Shimada E Mazda T Takanashi M Kawaguchi K. Immunoglobulin E Oligomers Identified in Blood Components Activate Mast Cells: Relevance to Anaphylactic Transfusion Reaction. Transfusion (2011) 51(11):2327–36. 10.1111/j.1537-2995.2011.03126.x 21470237

[11] Galli SJ Tsai M. Ige and Mast Cells in Allergic Disease. Nat Med (2012) 18(5):693–704. 10.1038/nm.2755 22561833 PMC3597223

[12] Redegeld FA Yu Y Kumari S Charles N Blank U. Non-Ige Mediated Mast Cell Activation. Immunol Rev (2018) 282(1):87–113. 10.1111/imr.12629 29431205

[13] Mukai K Tsai M Saito H Galli SJ. Mast Cells as Sources of Cytokines, Chemokines, and Growth Factors. Immunol Rev (2018) 282(1):121–50. 10.1111/imr.12634 PMC5813811 29431212

[14] Carroll-Portillo A Surviladze Z Cambi A Lidke DS Wilson BS. Mast Cell Synapses and Exosomes: Membrane Contacts for Information Exchange. Front Immunol (2012) 3:46. 10.3389/fimmu.2012.00046 22566928 PMC3342342

[15] Galli SJ Kalesnikoff J Grimbaldeston MA Piliponsky AM Williams CM Tsai M. Mast Cells as “Tunable” Effector and Immunoregulatory Cells: Recent Advances. Annu Rev Immunol (2005) 23:749–86. 10.1146/annurev.immunol.21.120601.141025 15771585

[16] Shefler I Salamon P Hershko AY Mekori YA. Mast Cells as Sources and Targets of Membrane Vesicles. Curr Pharm Des (2011) 17(34):3797–804. 10.2174/138161211798357836 22103851

[17] Tang K Liu J Yang Z Zhang B Zhang H Huang C. Microparticles Mediate Enzyme Transfer From Platelets to Mast Cells: A New Pathway for Lipoxin A4 Biosynthesis. Biochem Biophys Res Commun (2010) 400(3):432–6. 10.1016/j.bbrc.2010.08.095 20801099

[18] Shefler I Salamon P Reshef T Mor A Mekori YA. T Cell-Induced Mast Cell Activation: A Role for Microparticles Released From Activated T Cells. J Immunol (2010) 185(7):4206–12. 10.4049/jimmunol.1000409 20810987

[19] Becker M Lemmermann NA Ebert S Baars P Renzaho A Podlech J. Mast Cells as Rapid Innate Sensors of Cytomegalovirus by TLR3/TRIF Signaling-Dependent and -Independent Mechanisms. Cell Mol Immunol (2015) 12(2):192–201. 10.1038/cmi.2014.73 25152077 PMC4654297

[20] Kulka M Alexopoulou L Flavell RA Metcalfe DD. Activation of Mast Cells by Double-Stranded RNA: Evidence for Activation Through Toll-Like Receptor 3. J Allergy Clin Immunol (2004) 114(1):174–82. 10.1016/j.jaci.2004.03.049 15241362

[21] Elayeb R Tamagne M Bierling P Noizat-Pirenne F Vingert B. Red Blood Cell Alloimmunization is Influenced by the Delay Between Toll-Like Receptor Agonist Injection and Transfusion. Haematologica (2016) 101(2):209–18. 10.3324/haematol.2015.134171 PMC4938341 26430173

[22] Caradec J Kharmate G Hosseini-Beheshti E Adomat H Gleave M Guns E. Reproducibility and Efficiency of Serum-Derived Exosome Extraction Methods. Clin Biochem (2014) 47(13–14):1286–92. 10.1016/j.clinbiochem.2014.06.011 24956264

[23] Fang X Liao R Yu Y Li J Guo Z Zhu T. Thrombin Induces Secretion of Multiple Cytokines and Expression of Protease-Activated Receptors in Mouse Mast Cell Line. Mediators Inflammation (2019) 2019:4952131. 10.1155/2019/4952131 PMC6878808 31814803

[24] Almizraq RJ Seghatchian J Acker JP. Extracellular Vesicles in Transfusion-Related Immunomodulation and the Role of Blood Component Manufacturing. Transfus Apher Sci (2016) 55:281–91. 10.1016/j.transci.2016.10.018 27865649

[25] Schwartz LB Min HK Ren S Xia HZ Hu J Zhao W. Tryptase Precursors are Preferentially and Spontaneously Released, Whereas Mature Tryptase is Retained by HMC-1 Cells, Mono-Mac-6 Cells, and Human Skin-Derived Mast Cells. J Immunol (2003) 170(11):5667–73. 10.4049/jimmunol.170.11.5667 12759448

[26] Fanning LB Boyce JA. Lipid Mediators and Allergic Diseases. Ann Allergy Asthma Immunol (2013) 111(3):155–62. 10.1016/j.anai.2013.06.031 PMC4088989 23987187

[27] Elieh Ali Komi D Shafaghat F Kovanen PT Meri S. Mast Cells and Complement System: Ancient Interactions Between Components of Innate Immunity. Allergy (2020) 75(11):2818–28. 10.1111/all.14413 32446274

[28] Boyce JA. Mast Cells and Eicosanoid Mediators: A System of Reciprocal Paracrine and Autocrine Regulation. Immunol Rev (2007) 217:168–85. 10.1111/j.1600-065X.2007.00512.x 17498059

[29] Schmidt AP Taswell HF Gleich GJ. Anaphylactic Transfusion Reactions Associated With Anti-Iga Antibody. N Engl J Med (1969) 280(4):188–93. 10.1056/NEJM196901232800404 4177970

[30] Koda Y Watanabe Y Soejima M Shimada E Nishimura M Morishita K. Simple PCR Detection of Haptoglobin Gene Deletion in Anhaptoglobinemic Patients With Antihaptoglobin Antibody That Causes Anaphylactic Transfusion Reactions. Blood (2000) 95(4):1138–43. 10.1182/blood.V95.4.1138.004k27_1138_1143 10666182

[31] Lambin P Le Pennec PY Hauptmann G Desaint O Habibi B Salmon C. Adverse Transfusion Reactions Associated With a Precipitating Anti-C4 Antibody of Anti-Rodgers Specificity. Vox Sang (1984) 47(3):242–9. 10.1111/j.1423-0410.1984.tb01592.x 6464422

[32] Cop N Ebo DG Bridts CH Elst J Hagendorens MM Mertens C. Influence of IL-6, IL-33, and TNF-Alpha on Human Mast Cell Activation: Lessons From Single Cell Analysis by Flow Cytometry. Cytom B (2018) 94(3):405–11. 10.1002/cyto.b.21547 28802100

[33] Yamaguchi M Sayama K Yano K Lantz CS Noben-Trauth N Ra C. Ige Enhances Fc Epsilon Receptor I Expression and Ige-Dependent Release of Histamine and Lipid Mediators From Human Umbilical Cord Blood-Derived Mast Cells: Synergistic Effect of IL-4 and Ige on Human Mast Cell Fc Epsilon Receptor I Expression and Mediator Release. J Immunol (1999) 162(9):5455–65. 10228025

[34] Abraham SN St John A. Mast Cell-Orchestrated Immunity to Pathogens. Nat Rev Immunol (2010) 10(6):440–52. 10.1038/nri2782 PMC4469150 20498670

[35] Virk H Arthur G Bradding P. Mast Cells and Their Activation in Lung Disease. Transl Res (2016) 174:60–76. 10.1016/j.trsl.2016.01.005 26845625

[36] Kempuraj D Selvakumar GP Thangavel R Ahmed ME Zaheer S Raikwar SP. Mast Cell Activation in Brain Injury, Stress, and Post-Traumatic Stress Disorder and Alzheimer’s Disease Pathogenesis. Front Neurosci (2017) 11:703. 10.3389/fnins.2017.00703 29302258 PMC5733004

[37] Cai C Cao Z Loughran PA Kim S Darwiche S Korff S. Mast Cells Play a Critical Role in the Systemic Inflammatory Response and End-Organ Injury Resulting From Trauma. J Am Coll Surg (2011) 213(5):604–15. 10.1016/j.jamcollsurg.2011.08.009 PMC3205312 21920785

[38] Dib M Zhao X Wang XD Andersson R. Role of Mast Cells in the Development of Pancreatitis-Induced Multiple Organ Dysfunction. Br J Surg (2002) 89(2):172–8. 10.1046/j.0007-1323.2001.01991.x 11856129

[39] Chang JC Leung J Tang T Holzknecht ZE Hartwig MG Duane Davis RD. Cromolyn Ameliorates Acute and Chronic Injury in a Rat Lung Transplant Model. J Heart Lung Transplant (2014) 33(7):749–57. 10.1016/j.healun.2014.03.004 PMC4336160 24768366

[40] Vlaar AP Juffermans NP. Transfusion-Related Acute Lung Injury: A Clinical Review. Lancet (2013) 382(9896):984–94. 10.1016/S0140-6736(12)62197-7 23642914

[41] Bogner V Stoecklein V Richter P Suren C Teupser D Kanz KG. Increased Activation of the Transcription Factor C-Jun by MAP Kinases in Monocytes of Multiple Trauma Patients is Associated With Adverse Outcome and Mass Transfusion. J Surg Res (2012) 178(1):385–9. 10.1016/j.jss.2011.12.035 22677613

[42] Biberthaler P Bogner V Baker HV López MC Neth P Kanz KG. Genome-Wide Monocytic Mrna Expression in Polytrauma Patients for Identification of Clinical Outcome. Shock (2005) 24(1):11–9. 10.1097/01.shk.0000163394.93467.77 15988315

[43] Mor A Shefler I Salamon P Kloog Y Mekori YA. Characterization of ERK Activation in Human Mast Cells Stimulated by Contact With T Cells. Inflammation (2010) 33(2):119–25. 10.1007/s10753-009-9165-8 19908133

[44] Wong CK Tsang CM Ip WK Lam CWK. Molecular Mechanisms for the Release of Chemokines From Human Leukemic Mast Cell Line (HMC)-1 Cells Activated by SCF and TNF-Alpha: Roles of ERK, P38 MAPK, and NF-Kappab. Allergy (2006) 61(3):289–97. 10.1111/j.1398-9995.2006.00972.x 16436136

[45] MohanKumar K Namachivayam K Song T Cha BJ Slate A Hendrickson JE. A Murine Neonatal Model of Necrotizing Enterocolitis Caused by Anemia and Red Blood Cell Transfusions. Nat Commun (2019) 10(1):3494. 10.1038/s41467-019-11199-5 31375667 PMC6677753

[46] Orinska Z Bulanova E Budagian V Metz M Maurer M Bulfone-Paus S. TLR3-Induced Activation of Mast Cells Modulates CD8+ T-Cell Recruitment. Blood (2005) 106(3):978–87. 10.1182/blood-2004-07-2656 15840693

[47] Ogaki S Taguchi K Maeda H Watanabe H Ishima Y Otagiri M. Kupffer Cell Inactivation by Carbon Monoxide Bound to Red Blood Cells Preserves Hepatic Cytochrome P450 Via Anti-Oxidant and Anti-Inflammatory Effects Exerted Through the HMGB1/TLR-4 Pathway During Resuscitation From Hemorrhagic Shock. Biochem Pharmacol (2015) 97:310–9. 10.1016/j.bcp.2015.07.035 26232728

[48] Shefler I Salamon P Mekori YA. Microrna Involvement in Allergic and Non-Allergic Mast Cell Activation. Int J Mol Sci (2019) 20(9):2145. 10.3390/ijms20092145 PMC6539777 31052286

[49] Almizraq RJ Norris PJ Inglis H Menocha S Wirtz MR Juffermans N. Blood Manufacturing Methods Affect Red Blood Cell Product Characteristics and Immunomodulatory Activity. Blood Adv (2018) 2:2296–306. 10.1182/bloodadvances PMC6156888 30217795

