# Supplementary material for: Exosomes From Packed Red Cells Induce Human Mast Cell Activation and the Production of Multiple Inflammatory Mediators
Source: Front Immunol. 2021 May 6;12:677905. doi: 10.3389/fimmu.2021.677905 (PMC8135094; doi:10.3389/fimmu.2021.677905)
Supplement: Supplementary file 1 [file DataSheet_1.docx]

**SUPPLEMENTARY INFORMATION**

**Title: Exosomes isolated from packed red cells induce human mast cell line activation and the production of multiple inflammatory mediators**

**Authors:** Xiaobin Fang1†, PHD. Jingyi Li2†, MD. Xuechao Hao3, MD. Weiyi Zhang4, MD. Jie Zhong 5, MD. Tao Zhu 6*, MD. Ren Liao 7* MD

**Correspondence Authors:**

Ren Liao, E-mail: liaoren7733@163.com.

Tau Zhu, E-mail: 739501155@qq.com

**Supplementary Data 1**.

**Supplementary methods: detailed method and material used in our study.**

**Isolation and** **identification of Exosome(EXs)：**Blood were collected into an EDTA tube (Greiner Bio-One) and centrifuged 3000 r ×15 min at 4℃. Platelet-free plasma (PFP) was separated and stored at -80℃ until used. EXs were extracted from PFP using ultracentrifugation. Briefly, PFP was centrifuged with 10,000 g for 30 minutes. The supernatant was transferred to a sterile vessel carefully. Then it was centrifuged at 170000×g for 120 minutes at 4 ℃. The supernatant was removed, and the cloudy material adhering to the tube wall was dissolved by sterile phosphate buffer saline (PBS) 100ul and stored at -80℃ (This protocol is presented in Supplement.2.). Characterization of EXs was identified by Transmission electron microscopy (TEM), Nanoparticle tracking analysis (NTA), and Western blot analysis(WB). **TEM:** EXs 50 μl was applied to the grid and negatively stained with 2 % phosphotungstic acid for 1 min. Transmission images of EXs were acquired by H-600 TEM (Hitachi, Tokyo, Japan). **NTA:** NTA was used to analyze the size and shape of EXs. 50 μl of EXs was diluted in 50 ml PBS, and NTA was performed using a ZETA VIEW instrument (PARTICLE METRIX) and the Zeta View Electrophoresis & Brownian Motion Video Analysis software. **Western blotting**: Exosome samples were subjected to SDS-PAG Eon 12% gels, followed by Western blotting. The following primary antibodies were used: rabbit anti-ALIX (abcam, 1:1,000), rabbit anti-CD81 (abcam, 1:2,000). Blots were visualized using HRP-conjugated secondary antibodies and the ECL Detection Reagent (Thermo Fisher Scientific) and were imaged on a LAS3000 image reader (Bio Rad, UK).

**Protein concentration of EXs:** EXs 50 μl were systematically lysed in 50 μl of RIPA buffer (Thermo Fisher Scientific Inc.) and sonicated in 4 °C water bath (10 s, 2 times). Protein concentration was then quantified using Pierce™ BCA Protein Assay kit (Thermo Fisher Scientific Inc.).

**Material:** HMC-1 were bought from Shanghai Cell Bank of Chinese Academy of Sciences (Shanghai, China). PD98059, SB203580, and SP600125, and TLR-3/dsRNA Complex were obtained from Merck-Millipore(Burlington, MA), poly (A: U) from Invivogen(Toulouse, France). Human antibody against ɑ-tubulin, ALIX, CD81, total ERK1/2, total P38, total JNK, Lamin B1, anti-TLR-3, anti-Tryptase-1, phospho-SAPK/JNK MAPK, phospho-P38 MAPK (Thr180/Tyr182), and phospho-ERK1/2 MAPK (p44/42) were purchased from Proteintech (Chicago, IL, USA) or Cell Signaling Technology (Beverly, MA, USA), or Abcam (Cambridge, UK). CCK-8 kit was obtained from Dojindo (Japan). ELISA Kit for IL-6, TNF-α, IL-4, CCL2 came from Neobioscience (Shenzhen, China). ELISA Kit for CXCL-1, CXCL-5, PAF, LTB-4, and VEGF from Abcam (Cambridge, UK). Antibiotics were purchased from Gibco BRL (Grand Island, NY, USA). All reagents in this study were analytical grade and available.

**Quantitative real-time PCR (qPCR):** Total RNA was captured by the TRizol agent, the Reverse transcription kit (Bio-Rad, Britain) was used to synthesize the cDNA. The expression analysis of cDNA was employed using Maxima™ SYBR Green qPCR Master Mix (Fermentas, Vilnius, Lithuania) as per the manufacturer's introduction. Relative mRNA expressions were standardized to 18s RNA. The qRT-PCR primers were listed in Supplement content.4.Table.2.

**Enzyme-linked immunosorbent assay (ELISA):** The concentrations of IL-6, TNF-α, IL-4, CCl-2, CXCL-1, CXCL-5, LTB-4, and VEGF in the supernatant were measured using ELISA according to the manufacturer's instruction. Mediators' concentrations are expressed as pg/mL of protein.

**Western blot analysis:** Cells in each group were collected and washed twice, mixed systematically in RIPA buffer, centrifuged with 13 000 g for 10 min. Total protein concentration was normalized using the BCA assay. Mixtures with 100 μg were electrophoresed on SDS-PAGE with a 10 % acrylamide gel and transmitted to the PVDF membrane After blocking for 1 h by 5 % milk, membranes were incubated at 4 ℃ overnight with human antibodies against lamin B1(proteintech, 1:1000), ɑ-tubulin (abcam, 1:2000), TLR-3 (abcam, 1:1000), Tryptase-1 (proteintech, 1:1000), phosphorylated-JNK (abcam, 1:1000), phosphorylated-P38 (Thr180/Tyr182) (Cell signaling technology, 1:1000), phosphorylated-ERK1/2(p44/42) (immunoway,1:5000), total JNK (proteintech,1:1000), P38 (proteintech, 1:3000), and ERK1/2 (affinity, 1:2000) overnight, followed with secondary antibodies (HRP-conjugated). Blots were visualized as the procedure above mentioned. The relative amount of protein was quantified by the ratio to ɑ-tubulin or lamin B1.

**Immunofluorescence:** HMC-1 was resuspended, and 100 µL solution was transferred into 96-well plates, which have been pretreated with polylysine for 30 min. HMC-1 were treated by PBS, EXs-nor, and EXs-RBC for 8 h and subsequently fixed with 4% paraformaldehyde for 10 min. Samples were blocked with donkey serum and incubated overnight with anti-Tryptase-1 antibodies, followed by donkey-anti-rabbit IgG (H+L) Highly Cross-Adsorbed secondary antibodies for 2 h. Nuclei were counterstained with DAPI. Images were visualized using an automatic positive fluorescence microscope (Zeiss, German).

**Supplementary Table 1**

Subject information in the study

| Blood origin | Normal volunteer | | | | RBC | | | |
| --- | --- | --- | --- | --- | --- | --- | --- | --- |
| Age/ blood type | 17 | 47 | 17 | 37 | O | A | B | AB |
| Sex | M | F | M | F | / | / | / | / |
| Storage time (d) | / | / | / | / | 12 | 12 | 11 | 13 |

Subject information in the study. Four volunteers were enrolled, and blood 8 ml were collected from each. Each 8ml from four RBC units with the type of "A", "B", "O", and "AB" were received from the operation room in West China Hospital. Different blood types were used to exclude the disturbance of blood type.

**Supplementary Table 2**

The primers used in our study.

| primers | Forward | Reverse |
| --- | --- | --- |
| TLR-3 | GCAGTCAGCAACTTCATGGC | GCAGTCAGCAACTTCATGGC |
| Tryptase-1 | GACAGGCTGGGGTAACATCG | GAAGGCAGAAGAATTGGGACTC |
| IL-6 | CAATATTAGAGTCTCAACCCCCAA | TCACCAGGCAAGTCTCCTCA |
| PGD-2 | CACCTACTCCGTGTCAGTGGTNF | AGCCCTGGGGAGTCCTATTG |
| TNF | CTCGAACCCCGAGTGACAAG | TGAGGTACAGGCCCTCTGAT |
| INF | AGCTCTGCATCGTTTTGGGT | CCTCACAGAGCAGAAGAACACA |
| IL-4 | CCTCACAGAGCAGAAGAACACA | TCCAACGTACTCTGGTTGGC |
| CCL-2 | GATCTCAGTGCAGAGGCTCG | TTTGCTTGTCCAGGTGGTCC |
| CXCL-1 | CGCCTCTGATCCAAGCCAC | CTGCACATAGTCCTGCACCA |
| CXCL-5 | GCCTGTTCTAGTCCTGGTGG | GGCATCTAAAAAGCTCAGCAATG |
| LTB-4 | CAGCTTGGCCCAGAAGGATG | AAGGTTGACTGCGTGGTAGG |
| PAF | GCGCGGGAAAGAGACCAATA | TCCTGCCTCTTCAGGAATCTG |
| VEGF | CTCACCAAGGCCAGCACATA | CCGGGATTTCTTGCGCTTTC |

The primers used for qPCR in our study.

**Supplementary Figure 1**


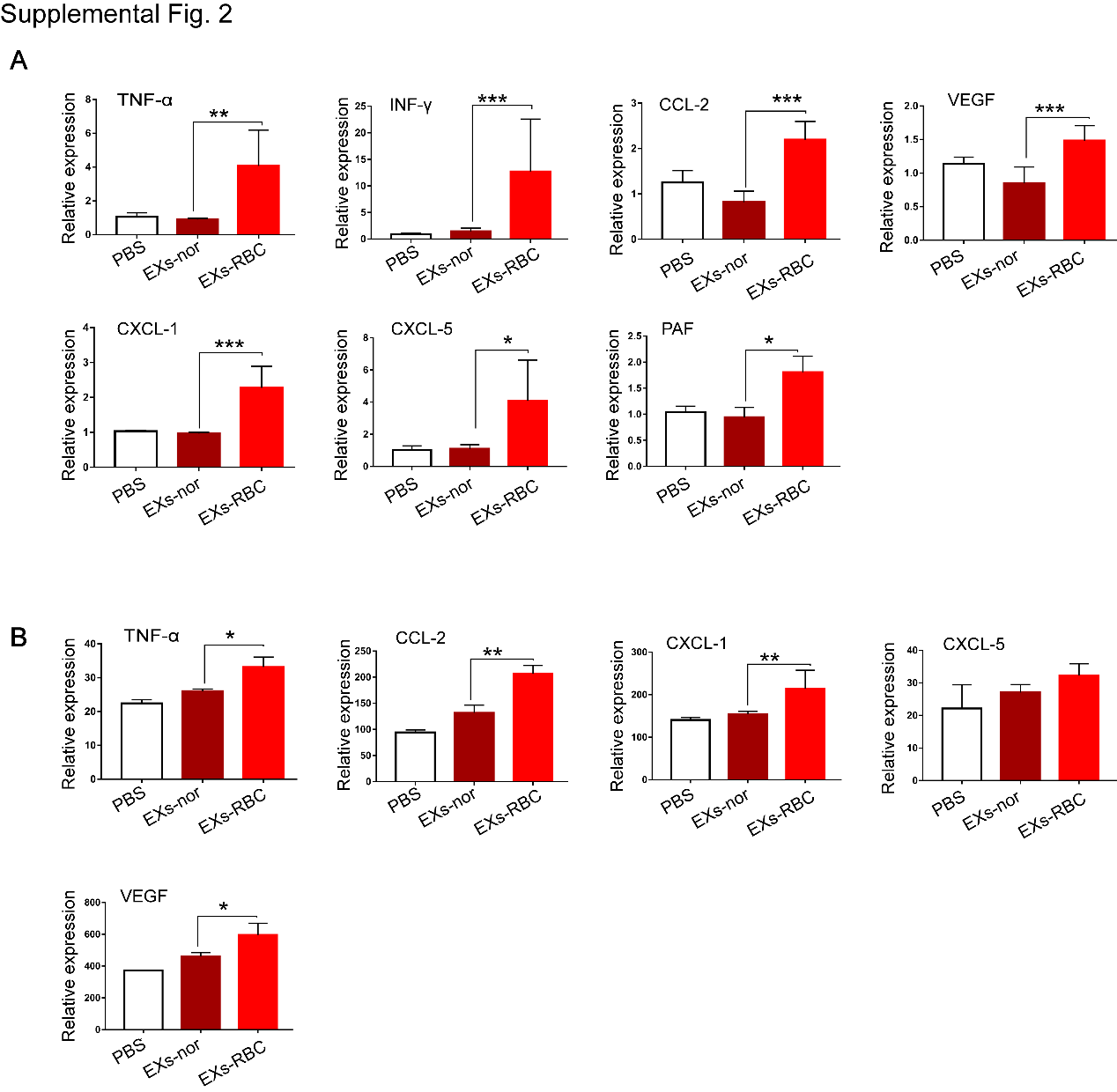


**Supplementary Figure 1 legend**

Increased expression and secretion of inflammatory mediators in HMC-1 after incubation with EXs-RBC. **(A)** HMC-1 was treated by PBS, EXs-nor, and EXs-RBC for 2 h. The level of INF-γ, CCL-2, CXCL-1, CXCL-5, PAF, and VEGF mRNA in HMC-1 raise significantly in the EXs-RBC group. **(B)** After 8 h treatment, the level of TNF-α, CCL-2, CXCL-1, and VEGF in supernatants significantly increased in the EXs-RBC group.

**Supplementary Figure 2**


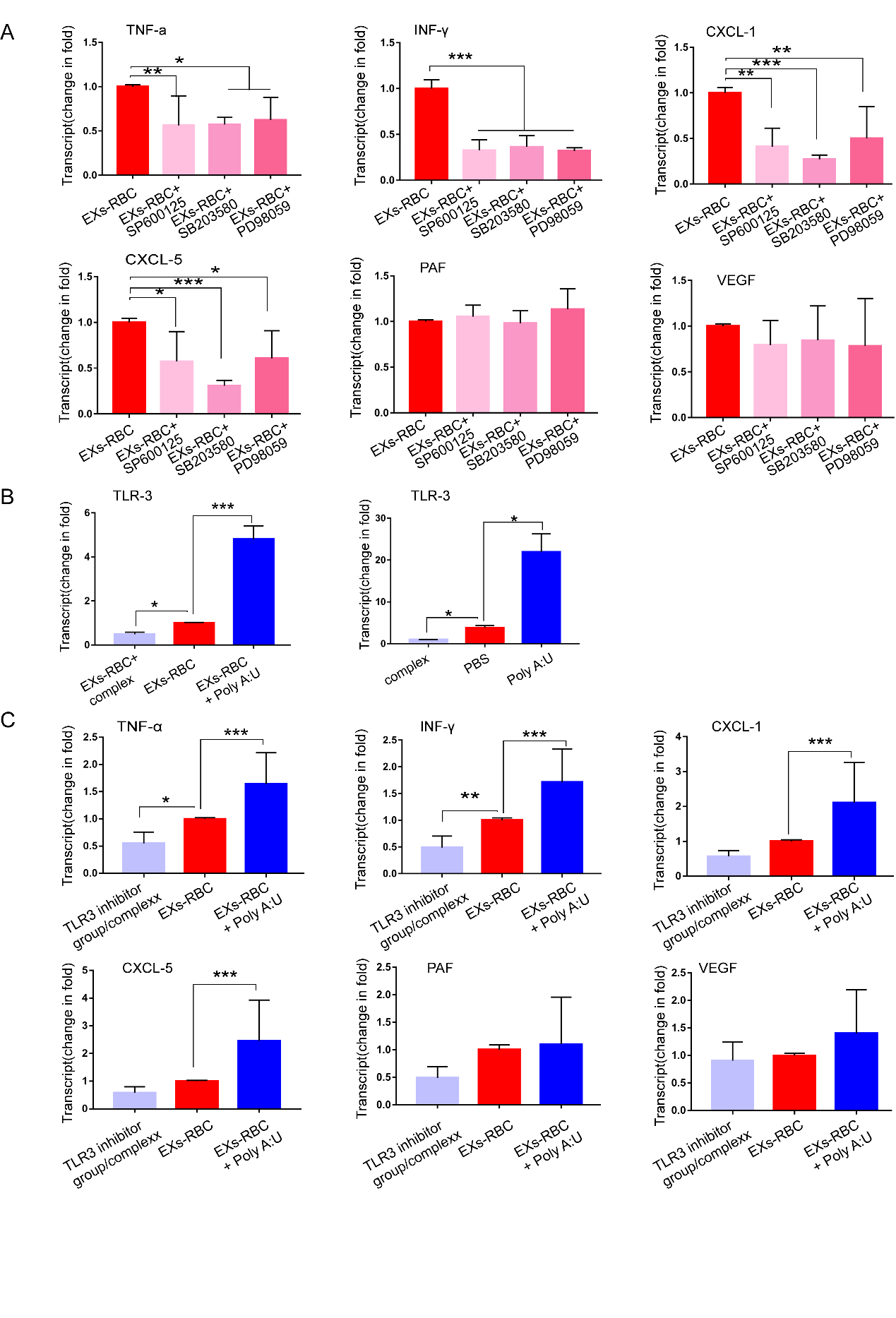


**Supplementary Figure 2 legend**

The effect of MAPKs inhibitors, TLR-3 agonist, and TLR-3 inhibitor on mediators expression in HMC-1 after incubation with EXs-RBC. **(A)** Expressions of TNF-α, IL-4, INF-γ, CXCL-1, and CXCL-5 but not PAF and VEGF in HMC-1 when stimulated with EXs-RBC. **(B)** The efficiency of TLR-3 agonist and TLR-3 inhibitor. Right: HMC-1 was incubated with EXs-RBC, EXs-RBC plus poly (A: U) 10 uM, and EXs-RBC plus TLR-3/dsRNA Complex 50 uM(left picture). Left: HMC-1 was incubated with PBS, poly (A: U) 10 uM, and TLR-3/dsRNA Complex 50 uM. With or without EXs-RBC stimulation, expressions of TLR-3 in HMC-1 increase in poly (A: U) 10 uM group, and decrease in TLR-3/dsRNA complex. **(C)** TLR-3/dsRNA complex decrease, while poly (A: U) increase the expressions of TNF-α, INF-γ, CXCL-1, CXCL-5, and LTB-4 but not of PAF and VEGF in HMC-1 when stimulated with EXs-RBC.
